# Frequent *NRG1* fusions in Caucasian pulmonary mucinous adenocarcinoma predicted by Phospho-ErbB3 expression

**DOI:** 10.18632/oncotarget.23800

**Published:** 2018-01-03

**Authors:** Domenico Trombetta, Paolo Graziano, Aldo Scarpa, Angelo Sparaneo, Giulio Rossi, Antonio Rossi, Massimo Di Maio, Davide Antonello, Andrea Mafficini, Federico Pio Fabrizio, Maria Carmina Manzorra, Teresa Balsamo, Flavia Centra, Michele Simbolo, Angela Pantalone, Michela Notarangelo, Paola Parente, Maria Cecilia Lucia Dimitri, Antonio Bonfitto, Fabiola Fiordelisi, Clelia Tiziana Storlazzi, Alberto L'Abbate, Marco Taurchini, Evaristo Maiello, Vito Michele Fazio, Lucia Anna Muscarella

**Affiliations:** ^1^ Laboratory of Oncology, IRCCS Casa Sollievo della Sofferenza Hospital, San Giovanni Rotondo, Foggia, Italy; ^2^ Unit of Pathology, IRCCS Casa Sollievo della Sofferenza Hospital, San Giovanni Rotondo, Foggia, Italy; ^3^ ARC-NET Research Centre and Department of Diagnostics and Public Health, Section of Pathology, University and Hospital Trust of Verona, Verona, Italy; ^4^ Division of Anatomic Pathology, Regional Hospital Umberto Parini, Aosta, Italy; ^5^ Oncology Department, IRCCS Casa Sollievo della Sofferenza Hospital, San Giovanni Rotondo, Foggia, Italy; ^6^ Department of Oncology, University of Turin, A. O. Ordine Mauriziano, Torino, Italy; ^7^ Department of Biology, University of Bari "A. Moro", Bari, Italy; ^8^ Unit of Thoracic-Oncology, IRCCS Casa Sollievo della Sofferenza Hospital, San Giovanni Rotondo, Foggia, Italy

**Keywords:** NRG1, pErbB3, mucinous adenocarcinoma, lung cancer, target therapy

## Abstract

*NRG1* fusions were recently reported as a new molecular feature of Invasive Mucinous Adenocarcinoma (IMA) of the lung. The *NRG1* chimeric ligand acts as a strong inductor of phosphorylation and tyrosine kinase activity of the ErbB2/ErbB3 heterodimer, thus enhancing the PI3K–AKT/MAPK pathways. The *NRG1* fusions were widely investigated in Asian IMA cohorts, whereas just anecdotal information are available about the occurrence of *NRG1* fusions in IMA Caucasian population.

Here we firstly explored a large Caucasian cohort of 51 IMAs and 34 non-IMA cases for the occurrence of NRG1 rearrangements by fluorescent in situ hybridization (FISH) and RNA target sequencing. FISH results were correlated to the immunohistochemical expression of phosphorylated-ErbB3 (pErbB3) receptor and the mutational status of *KRAS*, *EGFR* and *ALK* genes.

The *NRG1* rearrangements were detected in 31% IMAs and 3% non-IMAs and the CD74-*NRG1* fusion transcript variant was characterized in 4 *NRG1*-positive IMAs. Moreover, pErbB3 expression was found to be strictly associated to the mucinous pattern (*p* = 0.012, Chi-square test) and all IMA cases showing aberrant expression of pErbB3 demonstrated *NRG1* rearrangements. No significant correlation between *NRG1* rearrangements and *EGFR*, *KRAS* or *ALK* mutations respectively, was observed.

We report for the first time that *NRG1* fusions are driver alterations clearly associated with mucinous lung adenocarcinoma subtype of Caucasian patients and not exclusive of Asiatic population. pErbB3 immunostaining may represent a strong predictor of NRG1 fusions, pointing out the detection of pErbB3 by IHC as a rapid and effective pre-screening method to select the *NRG1*-positive patients.

## INTRODUCTION

The aberrant activation of members of the family of epidermal growth factor receptor tyrosine kinases (EGFR/ERBB) has been implicated in cancer development and progression. In lung cancer, mutations of epidermal growth factor receptor (*EGFR*) are a predictive marker of response to tyrosine kinase inhibitors (TKIs). The overexpression of ErbB3 receptor is a marker of acquired resistance of lung cancer to gefitinib and lapatinib [1–3], and is related to *HER2* and *MET* genes point mutations or amplifications [4]. In the last years, the deregulation of ErbB2/ErbB3 machinery is also emerging as an oncogenic trait of lung adenocarcinoma with mucinous pattern, a tumor subtype that is otherwise associated with *KRAS* mutations [5]. *ALK* positive lung adenocarcinomas often exhibit mucinous features, although these are usually morphologically distinct from classic mucinous adenocarcinoma [6]. Mucinous lung adenocarcinoma accounts for 2% to 5% of all lung adenocarcinomas and is characterized by a unfavorable clinical course and/or for which no effective treatment exists [7, 8]. The chimeric *CD74-NRG1* (Cluster of Differentiation 74-Neuregulin-1) gene derived by the chromosome 8 rearrangement is recently reported as the first potentially treatable oncogenic driver alteration associated with this specific pattern of lung adenocarcinoma. *NRG1* rearrangements accounted for a large portion of Invasive Mucinous Adenocarcinoma (IMA) of the lung [9] of Asian patients, both in *KRAS*-wild type and *KRAS* mutated tumors. However, it is of note that quite all the just published study cohorts were from Asian population. Only three Caucasian cases of IMA have been investigated for *NRG1* rearrangements by fluorescent *in situ* hybridization (FISH) and resulted negative [10].

The chimeric *NRG1* transcripts enhance the ectopic expression of the neuronal NRG1 III-β3 isoform, one of ErbB3 ligands, and induce the phosphorylation of the kinase domain of ErbB3 receptor and its heterodimerization with ErbB2. The consequent aberrant tyrosine kinase activity results in tumorigenesis via PI3K–AKT and MAPK pathways. Interestingly, the immunohistochemical expression of phospho-ErbB3 (pErbB3) has been occasionally reported to be associated with *NRG1* fusion-positive cases [9]. Such finding strongly suggests that pErbB3 immunostaining could represent an easy indirect approach to identify cancers carrying a *NRG1* fusion.

The aim of this study was to assess the prevalence of *NRG1* rearrangements in a Caucasian population of lung cancer patients, and verify the hypothesis that pErbB3 IHC overexpression is a predictive marker of *NRG1* fusions. We analyzed a cohort of 85 formalin-fixed paraffin-embedded (FFPE) IMA and non-IMA lung cancers from Italian patients by combining pErbB3 IHC and *NRG1* status assessed by FISH. Results were correlated with *KRAS*, *EGFR* and *ALK* genetic alterations.

## RESULTS

### pErbB3 expression in primary lung adenocarcinoma

**Figure 1:**
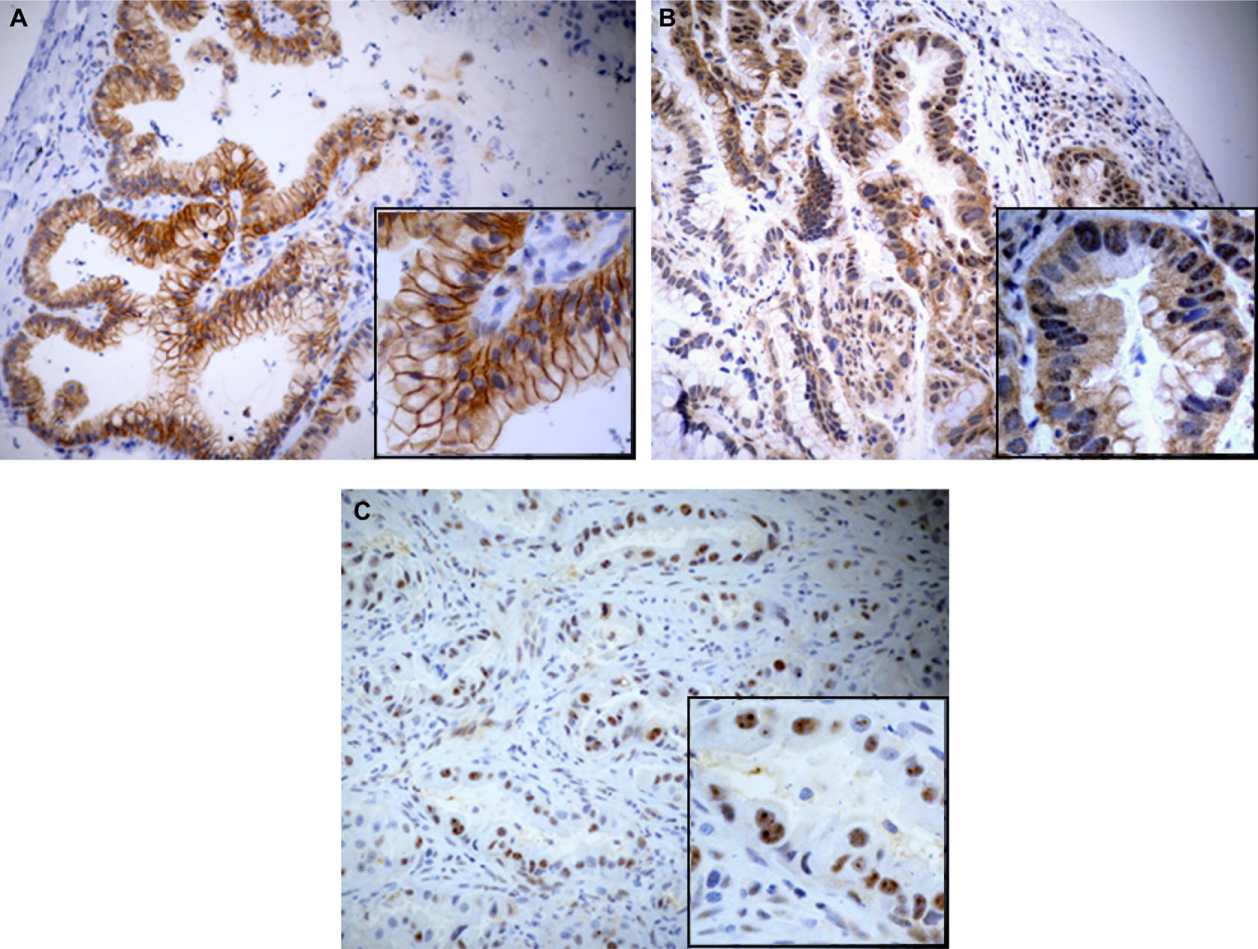
Microphotograph show mainly a membranous, and/or cytoplasmic positivity for pErbB3 antibody in IMA (A–B) Nuclear pattern respectively in IMA (B) and non-IMA (**C**) can be also observed.

Selective IHC was performed by staining representative tumor tissue sections of all 85 cases using a specific pErbB3 antibody (clone pTyr1289). Mainly a membranous, and/or cytoplasmic positivity for pErbB3 antibody was observed; only in two cases a nuclear pattern was noticed (Figure 1). An unambiguous difference in pErbB3 expression between IMA versus non-IMA group was found. More specifically, 15/51 (29%) IMA and 3/34 (9%) non-IMA samples cases resulted positive for pErbB3. Moreover, pErbB3 expression was found to be strictly associated to the mucinous pattern in a statistically significant manner (*p* = 0.012, *Chi-square test*).

### NRG1 rearrangement identification by Fluorescent *In Situ* Hybridization (FISH) analysis

**Figure 2:**
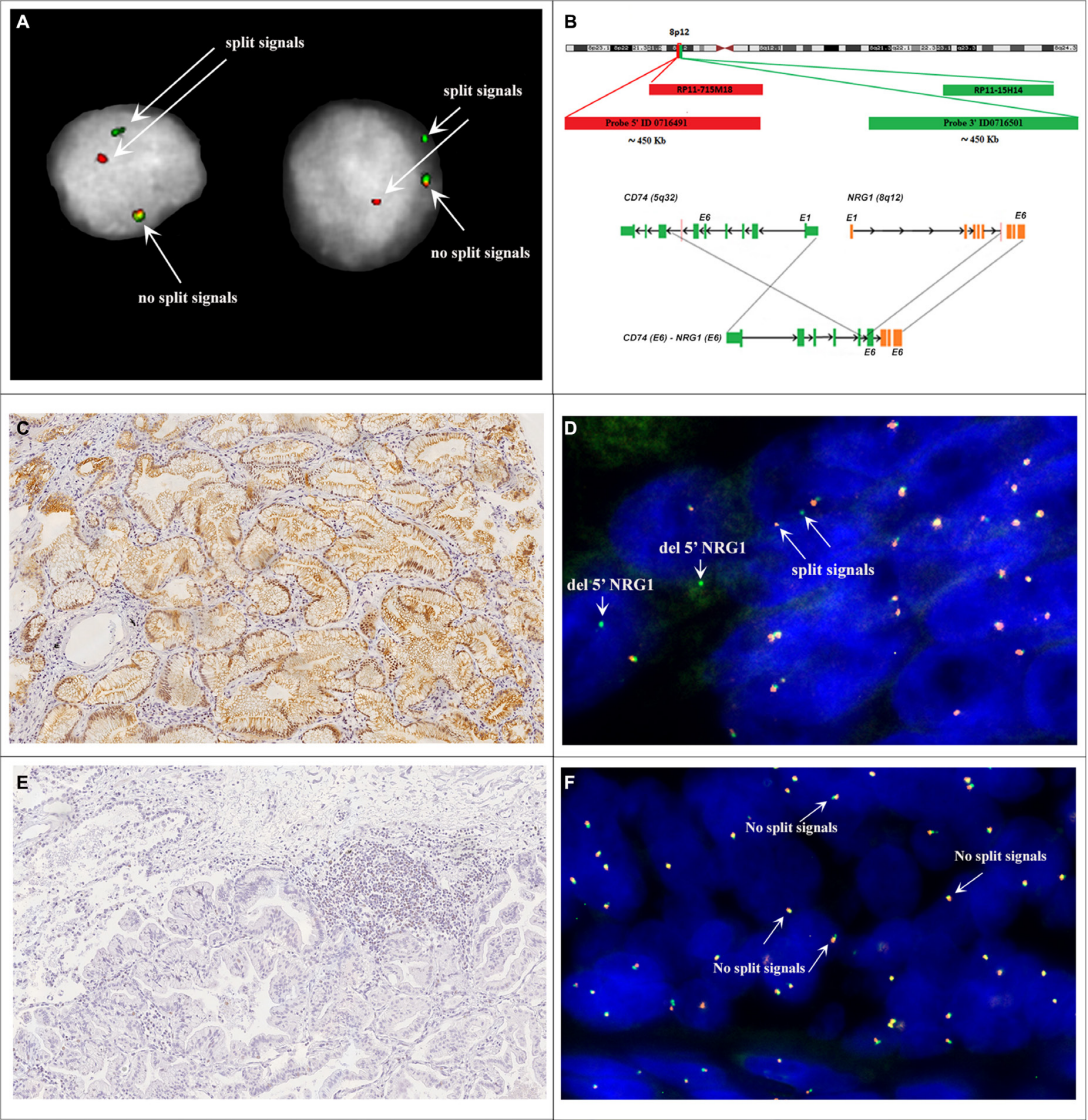
(**A**) Nuclei MD-175. (**B**) Schematic illustration of FISH probes map used for detection of *NRG1* rearrangement. For break-apart FISH assay telomeric BAC RP11-715M18 (chr8:32,264,921-32,433,449) and centromeric RP11-15H14 (chr8:32,679,447-32,840,717) were co-hybridized. Further FISH tests were performed by use of custom 5′-Red ID 0716491 (∼450 Kb) and 3’-Green ID 0716501 (∼450 Kb) probes. *NRG1* gene, BACs and custom probes are represented not in scale. (**C**) IMA (case MD-55) showing p-ErbB3 immunoreactivity. Magnification X20. (**D**) FISH analysis of case MD-55 shows disrupted *NRG1* locus. Red arrows highlight the split of the green and red probe signals (hybridizing to 3′ and 5′ regions of *NRG1* respectively). (**E**) IMA (case LCCH-556) without p-ErbB3 immunoreactivity. Magnification X20. (**F**) FISH analysis of case LCCH-556 shows the wild-type *NRG1* locus.

**Figure 3:**
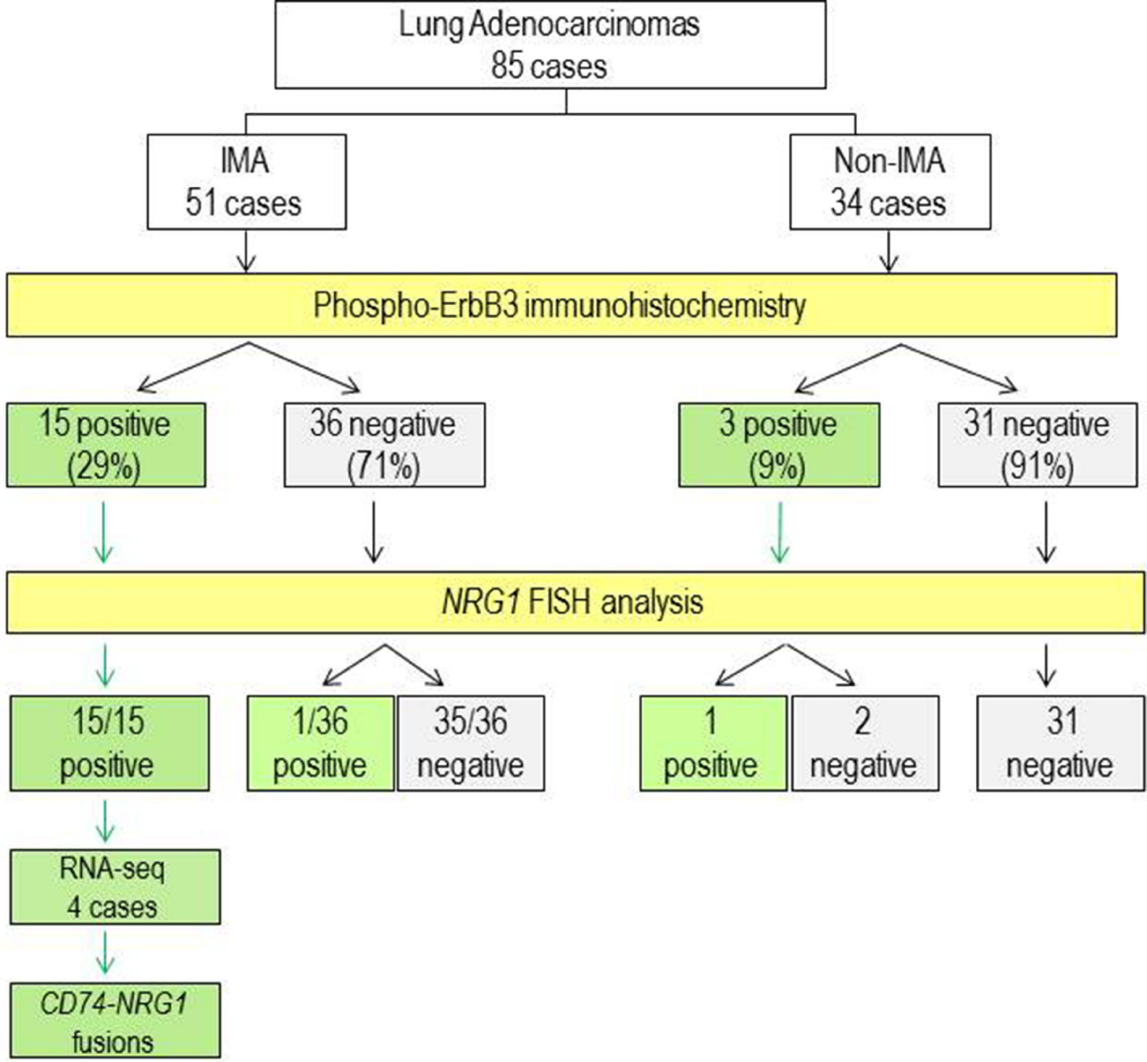
Flow chart of the study and summary of results IMA, invasive mucinous adenocarcinoma. White boxes, type and number of cases. Yellow boxes, analysis performed. Green boxes, positive results. Grey boxes, negative results.

*NRG1* rearrangements were assessed by FISH using two different probe sets: two BACs (Bacterial Artificial Chromosomes) hybridizing on 5′ and 3′ of the *NRG1* gene (RP11-715M18 and RP11-15H14), and custom fluorescent oligo-probes (Agilent Technologies) designed on flanking regions of *NRG1* (5′-Red ID0716491 and 3′-Green ID0716501 probes) (Figure 2). Both BAC and oligonucleotide probe sets were validated using MB-MD-175-VII cell line that carries *NRG1* rearrangement [11]. The interpretation of FISH signals followed the same criteria used for *ALK* and *ROS1* rearrangements in non small cell lung cancers [12, 13]. Briefly, samples were considered positive if at least 15% (of a minimum of 50 valuable nuclei) of cells showed split *NRG1* 5′ and 3′ probe signals or isolated 3′ signals. The distance between two separated signals was estimated using twice the size of the biggest signal size. As results, *NRG1* rearrangements were found in 16 of 51 (31%) IMA and only 1 of 34 (3%) non-IMA cases (Figure 3). The mean range of rearranged-positive cell rate in the *NRG1*-rearranged groups were 30% (range 18–55%). Both split signals and single 3’ signals were observed indicating the co-occurring deletion of 5′ portion of *NRG1* gene and the number of split was more higher than isolated 3′ signals.

### NRG1 fusion transcript characterization

Genetic rearrangements does not necessarily prove expression of a fusion gene, so we searched for the presence of an *NRG1* transcript fusion in the FISH positive samples. We screened by RNA target sequencing 4 *NRG1* FISH-positive IMAs for which the RNA from FFPE was available. We used a customized RNA-seq panel to target the described fusion transcripts of *NRG1* gene *CD74*(Ex6)-*NRG1*(Ex6), *CD74*(Ex8)-*NRG1*(Ex6), *SLC3A2*(Ex5)-*NRG1*(Ex6)). All four cases expressed the same *CD74(Ex6)-NRG1(Ex6)* fusion variant. As consequence of the rearrangement, an active chimeric ligand is predicted to be generated, which retains the Nrg1 βIII membrane-tethered EGF-like domain, as previously described (Figure 2).

### Correlation analysis between the presence of NRG1 rearrangement detected by FISH and immunopositivity of pErbB3

*NRG1* rearrangements were reported to produce an increase in the fusion transcript and chimeric ligand that aberrantly induces ErbB2/ErbB3 heterodimerization and ErbB3 receptor activation by phosphorylation. So, we expected to obtain a positive IHC results using an antibody against pErbB3 in tissue tumor samples where a functional *NRG1* rearrangement occurs.

In line to this hypothesis, results from pErbB3 immunostaining performed on all 85 FFPE lung cancer samples (51 IMAs and 34 non-IMAs) were correlated with the presence of *NRG1* rearrangements assessed by FISH. As results, of the 16 IMAs with *NRG1* rearrangement, 15 resulted pErbB3 positive and only one was negative, suggesting a sensitivity of 100% and a specificity of 97% of the indirect IHC immunohistochemical assay (Figure 3).

All adenocarcinomas with the *NRG1* fusions showed an increased expression of pErbB3 in tumor cells compared to the adjacent normal bronchial epithelium in which a basal level staining of the protein was demonstrated. Details were observed for the MD-10 and LCCH-584 cases. In the first one, the *NRG1* rearrangement was identified in 47% of pErbB3-positive area and in 10% of pErbB3-negative area. In MD-10 lung tissue, the *NRG1* rearrangement was found in 55% of IHC-positive area and in 4% of IHC-negative area.

### Correlation of NRG1 fusions with other molecular lesions of lung IMAs

The assessment of *EGFR* and *KRAS* mutations in the cohort is reported in Supplementary Table 4. *KRAS* mutations were more frequent in IMAs (18/46, 39%) than in non-IMAs (5/32, 16%) cases. Specific *KRAS* mutations observed in the IMAs included 7 G12D, 4 G12C, 3 G12V, 2 G12A and 1 G13D, whereas in non-IMAs 3 G12C, 1 G12V and 1 G12D were detected.

Interestingly, 6 of the 18 IMAs having a *KRAS* mutation also harbored the *NRG1* rearrangement (33%). All IMA cases were negative for *EGFR* mutations whereas exclusively 3 non-IMA cases resulted *EGFR* mutated. *ALK* rearrangement was detected just in one non-mucinous adenocarcinoma (data not shown) without coexisting *NRG1* fusions.

No significant correlation was observed between the presence of *NRG1* rearrangements or pErbB3 immunopositivity and *KRAS* mutations, patients’ age, gender, or tumor stage.

## DISCUSSION

The results of our study can be summarized as follows: i) *NRG1* rearrangements are firstly well described in a cohort of Caucasian lung adenocarcinoma patients and represent a feature of pulmonary IMA; ii) prevalence of *NRG1* fusions in Caucasian patients is similar to that reported in Asian population; iii) pErbB3 immunostaining is a strong predictor of *NRG1* fusions; iv) *NRG1* rearrangements are not mutually exclusive to *KRAS* mutations in IMAs also in Caucasian patients.

*NRG1* fusion genes in lung adenocarcinoma have been reported for the first time in 4 of 15 (27%) Asiatic IMAs [9]. Very few other studies confirmed this finding, but exclusively on Asian cohorts, and reported a prevalence of *NRG1* fusions ranging from 8% to 27% [14–16]. Only 3 Caucasian IMA cases were analyzed to date, but *NRG1* rearrangements were not detected by FISH (Table 1) [10]. Our finding of a 31% (16/51 cases) prevalence of *NRG1* rearrangements in the largest cohort of Caucasian lung IMAs analyzed to date is the first extensive analysis reported and overlaps the prevalence described in Asian lung cancer patients. These data support a link between *NRG1* fusions and lung mucinous adenocarcinoma, regardless of ethnical or geographical origin. However, we found that the *NRG1* fusion is not exclusive of mucinous lung adenocarcinomas but it can be also found with a very lower prevalence (3%) in non-IMA lung population. Similarly to the other lung cancer gene fusions, we firstly reported *NRG1* rearrangement patterns in form of isolated 3′ signals, thus indicating a strength analogy with *ALK* and *ROS1* fusions, where a 5′ gene deletion was frequently observed.

**Table 1:** Prevalence of *NRG1* fusions reported in invasive mucinous adenocarcinomas of the lung

Study (References)	Ethnicity	NRG1-fusion^*^	KRAS mutation	KRAS mutation plus NRG1 fusion	Tissue
Fernandez-Cuesta et al. (2014)	Asian (Japan)	4/15 (27%)	6/15 (40%)	0/4 (0%)	frozen
Gow et al. (2014)	Asian (Taiwan)	1/13 (8%)	3/12 (25%)	0/1 (0%)	frozen
Nakaoku et al. (2014)	Asian (Japan)	6/90 (7%)	56/90 (62%)	0/6 (0%)	frozen
Shin et al. (2016)	Asian (Korea)	16/59 (27%)	29/59 (49%)	10/16 (62%)	frozen
Duruisseaux et al. (2016)	Caucasian (3) Asian (1) North African (1)	1/5 (20%)^a^	0/5 (0%)	0/5 (0%)	FFPE
*This Study*	*Caucasian (Italy)*	*16/51 (31%)*	*18/46 (39%)*	*5/46 (11%)*	*FFPE*

^*^The prevalence of *NRG1* fusions is calculated on the effective number of IMAs evaluated for this aberration.

^a^The case showing *NRG1* rearrangement was Asian (Vietnamese).

FFPE, formalin-fixed paraffin-embedded.

Our RNA sequencing of four *NRG1* rearranged IMA cases identified the same *CD74-NRG1* chimeric transcript, thus corroborating our FISH results. Indeed, *CD74* has been reported as the most frequent *NRG1* fusion partner, but *SLC3A2, VAMP2, RBPMS, WNR* and *SDC4* may be alternative 5′ partners of the chimeric gene [16–18]. All these *NRG1* fusions are predicted to retain the EGF-like domain of the wild-type *NRG1* III-β3 isoform. The free active NRG1 chimeric ligands in the extracellular spaces bind their the pErbB3 receptor in an autocrine or paracrine fashion and produces oncogenic signals through ErbB2-ErbB3 heterodimers that leads to phosphorylation of ErbB3 [9]. We thus hypothesized that the expression of phosphorylated ErbB3 could be a potential marker of *NRG1* fusion genes.

Our data show that the expression of pErbB3 as detected by immunohistochemistry is a feature of one third (16/51, 31%) of the IMA subtype of lung cancer (*p* = 0.002, *Chi-square test*). On immunohistochemical analysis, pErbB3 was found localized mainly in the cell membrane and cytoplasm of tumor cells and only in two cases in the nucleus. There are few studies regarding the localization of pErbB3. These studies suggest that ErbB3 localized in the cell membrane translocates partially to the nucleus via ligand stimulation where it targets a particular gene to induce the expression of specific oncoproteins [19]. Of particular interest, we found that the genomic rearrangements of *NRG1* were identified by FISH analysis in all the 15 IMA pErbB3 immunopositive cases and in one of three non-IMA pErbB3 immunopositive cases. Notably, a *NRG1* rearrangement was found in only one of the 36 pErbB3 immunonegative IMAs.

These data suggest a high sensitivity and specificity of pErbB3 immunoreactivity as a strong predictor of *NRG1* fusion genes identified by FISH analysis on FFPE tissues. To date, *NRG1* fusions were identified quite exclusively by RNA sequencing and just in one case confirmed by FISH analysis. No pre-selection by p-ErbB3 overexpression was reported in any studies. Our approach should be much more user-friendly compared to a Next Generation Sequencing due to the high costs and the quality of samples required for this type of analysis.

Finally, taking into account the prevalence of *KRAS* mutations in the lung mucinous subtypes [20], we investigated the relationship between *NRG1* fusion and *KRAS* mutations. Although it has been suggested that these two lesions could be mutually exclusive each other in IMAs [9, 14], either our data in Caucasian patients and those reported by Shin et al. in Korean patients [16] refuted this hypothesis (see Table 2). We found that six of the 16 tumors showing *NRG1* fusion also harbored a mutations in *KRAS* gene, proposing an heterozygous nature of the fusion alleles in these samples and also declining the previous reported suggestion to use the presence of *KRAS* mutations as an exclusion criteria to perform FISH analysis at *NRG1* locus [10]. *NRG1* fusion coexisting with *EGFR* or *ALK* fusion were not identified.

**Table 2:** Clinical and pathological features of lung adenocarcinoma patients enrolled for the study (*n* = 85)

Characteristics	*n* (%)
Age at diagnosis (mean ± SD)	65 ± 9
Sex	
M	53 (64%)
F	32 (36%)
Adenocarcinoma subtype	
Mucinous	51 (60%)
non-mucinous	34 (40%)
Tumour Stage (pT)	
1a	16 (19%)
1b	12 (14%)
2a	20 (24%)
2b	13 (15%)
3	11 (13%)
4	6 (7%)
x	7 (8%)
Lymph nodes stage (pN)	
0	75 (88%)
1	3 (4%)
x	7 (8%)
Metastasis stage (pM)	
M0	71 (84%)
M1	5 (6%)
x	9 (10%)
UICC Stage	
IA	26 (31%)
IB	19 (22%)
IIA	13 (15%)
IIB	11 (13%)
IIIA	3 (4%)
IIIB	0 (0%)
IV	5 (6%)
Missed	8 (9%)

The translational impact of the *NRG1* fusions arises from the following recently published functional investigations. It has been shown that cancer cells harboring *NRG1* fusions overexpress the EGF-like domain of NRG1 III-β3 as a critical step for lung cancer tumorigenesis [9, 14, 16–18]. The expression of *CD74-NRG1* fusion gene is able to promote cancer stem cell properties and it is involved in stem cell function of several types of cancers, including lung cancer. This data implies the existence of a mechanism by which the activated ErbB receptors contribute to the acquisition of CSC (Cancer Stem Cell)-like characteristics together with the ability of cancer cells to develop a resistance to chemotherapy [21]. Of interest, NRG1 fusions have been proved to be coexistent with ALK fusion or RAS mutation in NSCLC patients both in primary and in metastatic sites of lung tumors [19, 22, 23] and it has been recently considered as a potential mechanism of resistance after treatment with crizotinib in ALK-rearranged NSCLC cell lines [24]. This assumption has been further confirmed showing that, under treatment with second-generation ALK inhibitors, NSCLC cells activated the EGFR family pathways directly through the NRG1-ErbB3-EGFR activation axis [25]. In support to these *in vitro* reports, the onset of the *SLC3A2-NRG1* gene fusion during the natural history of two invasive mucinous lung adenocarcinoma in Asiatic patients has been described. These heavily pre-treated patients received the combination of lumretuzumab, a monoclonal anti-ErbB3 antibody, plus erlotinib, an anti-EGFR small molecule, showing tumor shrinkage [26].

The aberrant tyrosine kinase activity of ErbB2/ErbB3 receptors caused by *NRG1* fusions represent an opportunity for the development of new anticancer drugs acting through inhibition of the ErbB network. This has already been shown to be a successful approach with EGFR and ErbB2 inhibitors [27–28]. To date, ErbB3 specific up-regulation has not been yet targeted by drugs showing a clear clinical activity. Monoclonal antibodies that target different sites of ErbB2 and ErbB3 domains and block the heterodimerization process are actually under evaluation [29]. Many of these clinical trials are biomarker-selected randomized studies that involve lung cancer patients and assess exclusively the status of ErbB2 receptor by mutation screening or immunohistochemistry (NCT01827267, NCT02289833, NCT02387216). As *NRG1* fusions induce the dimerization of ErbB3 receptor, and may thus represent a useful additional molecular marker to stratify patients and test the efficacy of these compounds.

In conclusion, our study documents that *NRG1* fusions are a feature of a subgroup of IMA of the lung, regardless of ethnicity of patients, and gives for the first time a clear evidence of a strong association between pErbB3 expression and *NRG1* fusions. This genomic alteration may represent a unique therapeutic opportunity to test the efficacy of compounds designed to inhibit ErbB network activity in this subset of IMA of the lung.

## MATERIALS AND METHODS

### Cell lines

MB-MD-175-VII breast cancer and H1993 lung cancer cell lines were purchased from the American Type Culture Collection (ATCC) and were cultivated at 37°C in DMEM/F12 and RPMI 1640 medium respectively, supplemented with 10% FBS and 1% penicillin and streptomycin.

### Patients and tissue samples

A total of 85 primary lung adenocarcinoma from Caucasian Italian patients (51 IMAs and 34 non-IMAs) were collected from 2002 to 2015 at the IRCCS “Casa Sollievo della Sofferenza” in San Giovanni Rotondo and IRCCS Arcispedale Santa Maria Nuova in Reggio Emilia as formalin fixed paraffin embedded (FFPE) blocks (Table 2).

The mean patient’s age at the time of diagnosis was 65 ± 9 (mean ± SD) with a range from 38 to 84 years. More than half of the patients were men (64%) versus 36% of women. All patients underwent curative surgery and all tumors were evaluated by two expert pulmonary pathologists (GR and PG) and classified as non-IMA and IMA according to the current WHO classification [30]. Representative blocks of the each neoplasm were selected for immunohistochemical and molecular studies. Materials and associated demographic and clinical information were collected according to the Local Ethics Committee approval (prot. 08/CE-52/CE IRCCS Casa Sollievo della Sofferenza. Prot. 2013/0026784 IRCCS Arcispedale Santa Maria Nuova in Reggio Emilia).

### Immunohistochemical analysis

Each lung cancer was characterized using the routinely diagnostic panel of antibodies reported in Supplementary Table 1 by an automated immunostainer (Benchmark XT, Ventana Medical System). Immunohistochemical expression of pErbB3 (clone Tyr1289, Cell Signaling) was performed on Autostainer Link 48 (Dako, Agilent) and FFPE cell pellets from the H1993 cell lines and tissue sections from breast cancers were used as positive controls. Briefly, after antigen retrieval in Tris-EDTA buffer (0.01 M) at 98°C, as indicated by the manufacturer, 3 µm FFPE tumor sections were incubated with hydrogen peroxide (0.3% v/v) in methanol for quenching endogenous peroxidase activity and thereafter incubated with primary antibody for 45 min at room temperature. The reaction was revealed using the EnVisionTMFLEX+ detection kit (Dako, Agilent). The sections were counterstained with Mayer’s hematoxylin and mounted with Biomount (BIO-OPTICA). As negative control, the primary antibody was replaced by isotype specific non-immune rabbit IgG.

After independent observation by two expert pathologists (GR and PG), pErbB3 protein expression was defined as positive if at least 10% of lung adenocarcinoma cells showed moderate or strong membrane and/or cytoplasmic reactivity [31].

### DNA and RNA extraction

Unstained FFPE cancer tissue sections (10µm thick) were microdissected to enrich for at least 60% cancer cells. DNA was extracted using the GeneRead DNA FFPE kit (Qiagen) and quantified with the Qubit dsDNA BR assay Kit (Life Technologies). DNA from cell lines was prepared using a standard Phenol-Chloroform procedure. Total RNA from FFPE sections was purified with the RecoverAll™ Total Nucleic Acid Isolation kit (Ambion). RNA from cell lines was extracted using Trizol Reagent (Life Technologies). The quality and quantity of the RNA was assessed using the Qubit RNA BR assay (Life Technologies).

### Fluorescence *in situ* hybridization (FISH) analysis

Structural rearrangements of *NRG1* gene (Chr 8p12) were assessed by FISH analysis with two different probe sets for break-apart assay: BAC and Oligonucleotide probe sets designed on flanking regions of the *NRG1* gene. BACs were selected according to the February 2009 release of GRCh37/hg19 by the University of California at Santa Cruz Human Genome Browser (http://genome.ucsc.edu), and were RP11-715M18 (chr8: 32,264,921-32,433,449) and RP11-15H14 (chr8: 32,679,447-32,840,717), encompassing the 5′-*NRG1* and 3′-*NRG1* respectively. The set of Oligonucleotide probes were designed (5’-Red ID0716491 and 3’-Green ID0716501 probes) using the Agilent Sure Design software (https://earray.chem.agilent.com/suredesign).

Three µm FFPE sections were hybridized with BAC probes labeled by nick translation. Briefly, 600 ng of labeled probes were used; hybridization was performed at 37°C in 2× saline-sodium citrate (SSC), 50% (vol/vol) formamide, 10% (wt/vol) dextran sulfate, 5 μg Cot-1 DNA (Bethesda Research Laboratories), and 3 μg sonicated salmon sperm DNA in a volume of 10 μl. Post-hybridization washings were at 73°C in 2× SSC for 2 min, followed by three washes in 2x SSC at room temperature. Nuclei were stained by 4,6-diamidino-2-phenylindole (DAPI). The hybridized slides were viewed on an Olympus IX-50 epifluorescence microscope (Olympus) equipped with a cooled CCD camera (Princeton Instruments) at 100X magnification with oil immersion. The GenASIs software (Applied Spectral Imaging, Carlsbad, CA, USA) was used to generate multicolor images. FISH with Agilent custom oligonucleotide probes were performed according to the manufacturer’s protocol. *NRG1* fusion rate was assessed by scoring all evaluable nuclei, in both pErbB3 positive and negative staining regions of the section. A minimum of 50 non-overlapping tumor cells with at least two each of 5′ and 3′ signals were examined for each case. Similar to *ALK* and *ROS1* FISH guidelines, the rearrangement-positive cells were defined as those with split signals or isolated red (3′) signals with a frequency ≥ 15% of the enumerated tumor cells.

### KRAS, EGFR and ALK mutations

DNA from lung cancers was analyzed for the *KRAS* and *EGFR* gene by Sanger sequencing and ddPCR (Supplementary Table 2). For Sanger sequencing, 50 ng of genomic DNA were amplified with appropriate primers, PCR products were purified using GFX PCR DNA and Gel Band Purification Kit (GE Healthcare). Sequencing reactions were performed using 5 ng of PCR products and Big Dye Terminator Ready Reaction mix v. 1.1 (Thermo Fisher), loaded on an ABI 3100 sequence detection system (Thermo Fisher) and analyzed using the Sequencing Analysis software v.3.7 (Thermo Fisher). Digital droplet PCR was performed using QX100™ Droplet Digital™ PCR System (Bio-Rad) and appropriate primers and probes for *KRAS* and *EGFR* mutations (www.bio-rad.com/it). Droplets were generated using a Droplet Generator and then amplified in a C1000 Touch™ deep-well Thermal Cycler (Bio-Rad). After cycling, the 96-well plate was placed into the QX200 Droplet Reader (Bio-Rad) where droplets of each sample are analyzed sequentially and fluorescent signals of each droplet are measured individually by a detector. Data were analyzed with Quanta Soft analysis software, version 1.7.4. Anaplastic lymphoma kinase (*ALK*) gene status was determined by immunohistochemical analysis using anti-ALK antibody (clone D5F3, Cell Signaling). All the adenocarcinomas showing cytoplasmic with /without membrane immunoreactivity were defined as ALK positive. The cases displaying weak (score 1) or moderate (score 2) ALK staining were also evaluated by FISH analysis. Adenocarcinomas demonstrating strong (3+) ALK immunoreactivity were defined as *ALK* rearranged.

### Fusion Transcript identification by RNA Target Sequencing

Total RNA (50 ng) was reverse transcribed using the SuperScript VILO cDNA Synthesis Kit followed by library generation using the Ion AmpliSeq Library Kit 2.0 and a Custom Ion AmpliSeq RNA Fusion Panel (ID IAD107474, Thermo Fisher Scientific) designed to target the described fusion transcripts of *NRG1* gene (*CD74(Ex6)*-*NRG1(Ex6)*, *CD74(Ex8)-NRG1(Ex6)*, *SLC3A2(Ex5)-NRG1(Ex6)*), additional 103 fusion transcripts and 12 housekeeping genes (*MYC*, *TBP*, *JUN*, *CFHR5*, *AFM*, *LMNA*, *MRPL13*, *MTTP*, *ITGB7*, *HBMS*, *APOB*, *LRP1*) as internal positive controls (Supplementary Table 3). Barcodes were added during library generation using the IonXpress Barcode Adapters. Libraries were quantified using the 2100 BioAnalyzer (Agilent Technologies), then pooled in equimolar concentrations. The library pool was templated using the Ion PGM Hi-Q Chef Kit on the Ion Chef and sequenced using an Ion 318 chip on the Ion Torrent PGM™ sequencer (Thermo Fisher Scientific). After sequencing, data were automatically transferred and analyzed on the Ion Reporter Server (Thermo Fisher Scientific) using the “single fusion” workflow in order to detect and annotate variants in *NRG1* fusion transcripts. This workflow aligns and counts reads matching with the target transcripts, taking into account only reads that overlap the theoretical target by more than 70%. Counts are normalized to the total number of mapped reads and expressed in reads per million.

### Statistical analysis

Study sample size was not calculated according to a pre-defined hypothesis, because the study was conducted with an explorative aim, in the absence of enough data available in the literature. Patients baseline characteristics have been reported as mean ± standard deviation (SD) or median ± range for continuous variables, and as frequencies and percentages for categorical variables. *NRG1*, *pErbB3* and *KRAS* status was compared between groups using Pearson Chi-square and Fisher’s exact tests, as appropriate.

## SUPPLEMENTARY MATERIALS TABLES






