# Supplementary material for: Frequent NRG1 fusions in Caucasian pulmonary mucinous adenocarcinoma predicted by Phospho-ErbB3 expression
Source: Oncotarget. 2018 Jan 3;9(11):9661–71. doi: 10.18632/oncotarget.23800 (PMC5839392; doi:10.18632/oncotarget.23800)
Supplement: Supplementary file 1 [file oncotarget-09-9661-s001.pdf]

Frequent *NRG1* fusions in caucasian pulmonary mucinous adenocarcinoma predicted by phospho-erbb3 expression

SUPPLEMENTARY MATERIALS

Supplementary Table 1: Monoclonal antibodies used for immunohistochemical profiling of lung cancer cohort

Antibody	Clone	Manufacturer	Source
Thyroid Transcription Factor-1	8G7G3/1	Ventana Medical System	Mouse
p40	BC28	Ventana Medical System	Mouse
CDX-2	EPR2764Y	Ventana Medical System	Rabbit
Cytokeratin 7	SP52	Ventana Medical System	Rabbit
Cytokeratin 20	SP33	Ventana Medical System	Rabbit
Napsin A	MRQ-60	Ventana Medical System	Mouse
pErbB3	Tyr1289	Cell Signaling Technology	Rabbit

Supplementary Table 2: Primer sequences and assays used for *KRAS* and *EGFR* mutational analysis

Primer name	Primer sequence (5′–3′)/ID assay	Methods
KRAS_ex2_F	CATGTTCTAAATATAGTCACA	Sanger Sequencing
KRAS_ex2_R	AACAAGATTTACCTCTATTG	Sanger Sequencing
KRAS Wt G12A	dHsaCP2000004	ddPCR
KRAS G12A	dHsaCP2000003	ddPCR
KRAS Wt G12C	dHsaCP2000008	ddPCR
KRAS G12C	dHsaCP2000007	ddPCR
KRAS Wt G12D	dHsaIS2503027	ddPCR
KRAS G12D	dHsaCP2000001	ddPCR
KRAS Wt G12V	dHsaCP2000006	ddPCR
KRAS G12V	dHsaCP2000005	ddPCR
KRAS Wt G13D	dHsaCP2000014	ddPCR
KRAS G13D	dHsaCP2000013	ddPCR
EGFR_Ex18_F	GCTGAGGTGACCCTTGTCTC	Sanger Sequencing
EGFR_Ex18_R	ACAGCTTGCAAGGACTCTGG	Sanger Sequencing
EGFR_Ex19_Ext_F	GCAATATCAGCCTTAGGTGCGGTC	Sanger Sequencing
EGFR_Ex19_Ext_R	CATAGAAAGTGAACATTTAGGATGTG	Sanger Sequencing
EGFR_Ex19_Int_F	CATGTGGCACCATCTCACA	Sanger Sequencing
EGFR_Ex19_Int_R	CCACACAGCAAAGCAGAAAC	Sanger Sequencing
EGFR_Ex20_F	CTCCCTCCAGGAAGCCTACGTGAT	Sanger Sequencing
EGFR_Ex20_R	TTTGCGATCTGCACACACCA	Sanger Sequencing
EGFR_Ex21_Ext_F	CTAACGTTCGCCAGCCATAAGTCC	Sanger Sequencing
EGFR_Ex21_Ext_R	GCTGCGAGCTCACCCAGAATGTCTGG	Sanger Sequencing
EGFR_Ex21_Int_F	CCTCACAGCAGGGTCTTCTC	Sanger Sequencing
EGFR_Ex21_Int_R	CCTGGTGTCAGGAAAATGCT	Sanger Sequencing
EGFR L858R	dHsaCP2000021	ddPCR
EGFR Wt L858R	dHsaCP2000022	ddPCR
EGFR L747_T751del	dHSACP2506752	ddPCR
EGFR Wt L747_T751del	dHSACP2506753	ddPCR
EGFR L747_S752del	dHSACP2506758	ddPCR
EGFR Wt L747_S752del	dHSACP2506759	ddPCR

*wt assays are labeled with FAM, Mutant assays are labeled with HEX.

Supplementary Table 3: Housekeeping and fusion transcripts included into the RNA-seq custom panel ID IAD107474. See Supplementary_Table_3

Supplementary Table 4: *NRG1, KRAS, EGFR* and *ALK* alterations in the lung adenocarcinomas study cohort. Supplementary_Table_4
