# Supplementary material for: Frequent NRG1 fusions in Caucasian pulmonary mucinous adenocarcinoma predicted by Phospho-ErbB3 expression
Source: Oncotarget. 2018 Jan 3;9(11):9661–71. doi: 10.18632/oncotarget.23800 (PMC5839392; doi:10.18632/oncotarget.23800)
Supplement: Supplementary file 2 [file oncotarget-09-9661-s002.docx]

**Supplementary Table 3.** Housekeeping and Fusion Transcripts included into the RNA-seq custom panel ID IAD107474.

**Nucleic Acid Type Transcript name**

RNA Housekeeping APOB

RNA Housekeeping AFM

RNA Housekeeping MRPL13

RNA Housekeeping LRP1

RNA Housekeeping ITGB7

RNA Housekeeping JUN

RNA Housekeeping HMBS

RNA Housekeeping LMNA

RNA Housekeeping TBP

RNA Housekeeping CFHR5

RNA Housekeeping MYC

RNA Housekeeping MTTP

RNA Fusion CD74-NRG1.C6N6

RNA Fusion CD74-NRG1.C8N6

RNA Fusion SLC3A2-NRG1.S5N6

RNA Fusion EML4-ALK.E13A20.COSF1062.1

RNA Fusion EML4-ALK.E13A20.COSF408

RNA Fusion EML4-ALK.E13A20.COSF410

RNA Fusion EML4-ALK.E13A20.COSF414

RNA Fusion EML4-ALK.E13A20.COSF462

RNA Fusion FUSION,EML4-ALK.E13A20.COSF489

RNA Fusion FUSION,EML4-ALK.E14A20.COSF1064

RNA Fusion EML4-ALK.E14A20.COSF1064.1

RNA Fusion EML4-ALK.E14A20.COSF1065

RNA Fusion EML4-ALK.E14A20.COSF413

RNA Fusion EML4-ALK.E14A20.COSF477.1

RNA Fusion EML4-ALK.E14A20.COSF491

RNA Fusion EML4-ALK.E15A20.COSF475

RNA Fusion EML4-ALK.E17A20.COSF1366.1

RNA Fusion EML4-ALK.E17A20.COSF1367.1

RNA Fusion EML4-ALK.E17A20.COSF1368

RNA Fusion EML4-ALK.E17A20.COSF1368.1

RNA Fusion EML4-ALK.E17A20.COSF732

RNA Fusion EML4-ALK.E17A20.COSF733

RNA Fusion EML4-ALK.E18A20.COSF487

RNA Fusion EML4-ALK.E18A20.COSF488

RNA Fusion EML4-ALK.E20A20.COSF409

RNA Fusion EML4-ALK.E20A20.COSF464

RNA Fusion EML4-ALK.E20A20.COSF490

RNA Fusion EML4-ALK.E20A20.COSF730.1

RNA Fusion EML4-ALK.E2A20.COSF478

RNA Fusion EML4-ALK.E2A20.COSF479.1

RNA Fusion EML4-ALK.E6A17

RNA Fusion EML4-ALK.E6A18

RNA Fusion EML4-ALK.E6A19.COSF1296

RNA Fusion EML4-ALK.E6A20.COSF411

RNA Fusion EML4-ALK.E6A20.COSF474

RNA Fusion EML4-ALK.E6A20.COSF493

RNA Fusion EML4-ALK.E6bA20.AB374362

RNA Fusion EML4-ALK.E7A20.NGS

RNA Fusion KIF5B-ALK.K15A19.COSF1061

RNA Fusion KIF5B-ALK.K15A20.COSF1060

RNA Fusion KIF5B-ALK.K15A20.COSF1381

RNA Fusion KIF5B-ALK.K17A20.COSF1257

RNA Fusion KIF5B-ALK.K24A20.COSF1058

RNA Fusion KLC1-ALK.K9A20.COSF1276

RNA Fusion KIF5B-RET.K15R11.COSF1255

RNA Fusion KIF5B-RET.K15R11.COSF1256

RNA Fusion KIF5B-RET.K15R12.COSF1232

RNA Fusion KIF5B-RET.K15R12.COSF1233

RNA Fusion KIF5B-RET.K15R12.COSF1237

RNA Fusion KIF5B-RET.K15R12.COSF1238

RNA Fusion KIF5B-RET.K15R12.COSF1239

RNA Fusion KIF5B-RET.K16R12.COSF1230

RNA Fusion KIF5B-RET.K16R12.COSF1231

RNA Fusion KIF5B-RET.K16R12.COSF1240

RNA Fusion KIF5B-RET.K16R12.COSF1240.1

RNA Fusion KIF5B-RET.K22R12.COSF1253

RNA Fusion KIF5B-RET.K22R12.COSF1254

RNA Fusion KIF5B-RET.K23R12.COSF1234

RNA Fusion KIF5B-RET.K23R12.COSF1235

RNA Fusion KIF5B-RET.K23R12.COSF1241

RNA Fusion KIF5B-RET.K24R11.COSF1262

RNA Fusion KIF5B-RET.K24R11.COSF1263

RNA Fusion KIF5B-RET.K24R7.COSF1242

RNA Fusion KIF5B-RET.K24R8.COSF1236

RNA Fusion CCDC6-RET.C1R12.COSF1271

RNA Fusion CCDC6-RET.C2R12.COSF1515

RNA Fusion CCDC6-RET.C8R11.COSF1518

RNA Fusion CD74-ROS1.C4R33.NGS

RNA Fusion CD74-ROS1.C6R32.COSF1202

RNA Fusion CD74-ROS1.C6R34.COSF1200

RNA Fusion SDC4-ROS1.S2R32.COSF1265

RNA Fusion SDC4-ROS1.S2R34

RNA Fusion SDC4-ROS1.S4R32.COSF1278

**RNA** Fusion SDC4-ROS1.S4R34.COSF1280

RNA Fusion SLC34A2-ROS1.S13R32.COSF1259

RNA Fusion SLC34A2-ROS1.S13R34.COSF1261

RNA Fusion SLC34A2-ROS1.S13R36

RNA Fusion SLC34A2-ROS1.S4R32.COSF1196

RNA Fusion SLC34A2-ROS1.S4R34.COSF1198

RNA Fusion EZR-ROS1.E10R34.COSF1267

RNA Fusion EZR-ROS1.E10R34.COSF1396

RNA Fusion TPM3-ROS1.T8R35.COSF1273

RNA Fusion LRIG3-ROS1.L16R35.COSF1269

RNA Fusion GOPC-ROS1.G4R35

RNA Fusion GOPC-ROS1.G4R36.COSF1188

RNA Fusion GOPC-ROS1.G4R36.COSF1243

RNA Fusion GOPC-ROS1.G8R35.COSF1139

RNA Fusion GOPC-ROS1.G8R35.COSF1140

RNA Fusion GOPC-ROS1.G8R35.COSF1210

RNA Fusion CEL-NTRK1.C7N7

RNA Fusion NFASC-NTRK1.N20N10

RNA Fusion TPR-ALK.T15A20

RNA Fusion HIP1-ALK.H21A20

RNA Fusion HIP1-ALK.H28A20

RNA Fusion CUX1-RET.C10R12

RNA Fusion MPRIP-NTRK1.M14N12

RNA Fusion MPRIP-NTRK1.M18N12

RNA Fusion MPRIP-NTRK1.M21N12

RNA Fusion MPRIP-NTRK1.M21N14

RNA Fusion CD74-NTRK1.C8N12

RNA Fusion TFG-NTRK1.T5N10.COSF1328

RNA Fusion TFG-NTRK1.T5N9

RNA Fusion TFG-NTRK1.T5N9.COSF1337

RNA Fusion TFG-NTRK1.T6N10

RNA Fusion SSBP2-NTRK1.S12N12

RNA Fusion SQSTM1-NTRK1.S5N10
