# Supplementary material for: Frequent NRG1 fusions in Caucasian pulmonary mucinous adenocarcinoma predicted by Phospho-ErbB3 expression
Source: Oncotarget. 2018 Jan 3;9(11):9661–71. doi: 10.18632/oncotarget.23800 (PMC5839392; doi:10.18632/oncotarget.23800)
Supplement: Supplementary file 3 [file oncotarget-09-9661-s003.docx]

**Supplementary Table 4.** *NRG1*, *KRAS*, *EGFR* and *ALK* alterations in the lung adenocarcinomas study cohort.

| **ID Sample** | **Adenocarcinoma** | ***NRG1*** | ***KRAS*** | ***EGFR*** | ***ALK*** |  |  |  |  |
| --- | --- | --- | --- | --- | --- | --- | --- | --- | --- |
|  | **subtype** |  |  |  |  |  |  |  |  |
| MD-1 | *mucinous* | wt | wt | wt | ne |  |  |  |  |
| MD-3 | *mucinous* | wt | mutated | wt | wt |  |  |  |  |
| MD-4 | *mucinous* | wt | wt | wt | wt |  |  |  |  |
| MD-6 | *mucinous* | wt | mutated | wt | wt |  |  |  |  |
| MD-7 | *mucinous* | wt | wt | ne | ne |  |  |  |  |
| MD-8 | *mucinous* | wt | wt | wt | wt |  |  |  |  |
| MD-9 | *mucinous* | wt | wt | wt | wt |  |  |  |  |
| MD-10 | *mucinous* | mutated | mutated | wt | wt |  |  |  |  |
| MD-11 | *mucinous* | mutated | mutated | wt | wt |  |  |  |  |
| MD-12 | *mucinous* | wt | wt | wt | wt |  |  |  |  |
| MD-13 | *mucinous* | wt | wt | wt | wt |  |  |  |  |
| MD-14 | *mucinous* | wt | mutated | wt | wt |  |  |  |  |
| MD-16 | *mucinous* | wt | ne | wt | wt |  |  |  |  |
| MD-17 | *mucinous* | wt | mutated | wt | wt |  |  |  |  |
| MD-18 | *mucinous* | mutated | mutated | wt | wt |  |  |  |  |
| MD-19 | *mucinous* | mutated | mutated | wt | wt |  |  |  |  |
| MD-20 | *mucinous* | wt | mutated | wt | wt |  |  |  |  |
| MD-21 | *mucinous* | wt | mutated | wt | wt |  |  |  |  |
| MD-25 | *mucinous* | wt | ne | ne | ne |  |  |  |  |
| MD-29 | *mucinous* | mutated | wt | wt | wt |  |  |  |  |
| MD-30 | *mucinous* | wt | wt | ne | ne |  |  |  |  |
| MD-31 | *mucinous* | wt | wt | wt | wt |  |  |  |  |
| MD-32 | *mucinous* | wt | wt | wt | wt |  |  |  |  |
| MD-33 | *mucinous* | wt | wt | wt | wt |  |  |  |  |
| MD-34 | *mucinous* | mutated | ne | ne | ne |  |  |  |  |
| MD-35 | *mucinous* | mutated | ne | ne | ne |  |  |  |  |
| MD-36 | *mucinous* | mutated | wt | wt | ne |  |  |  |  |
| MD-37 | *mucinous* | mutated | wt | wt | wt |  |  |  |  |
| MD-38 | *mucinous* | wt | wt | wt | wt |  |  |  |  |
| MD-39 | *mucinous* | wt | mutated | wt | wt |  |  |  |  |
| MD-40 | *mucinous* | wt | wt | wt | ne |  |  |  |  |
| MD-41 | *mucinous* | mutated | wt | wt | ne |  |  |  |  |
| MD-43 | *mucinous* | wt | wt | wt | wt |  |  |  |  |
| MD-44 | *mucinous* | wt | mutated | wt | wt |  |  |  |  |
| MD-45 | *mucinous* | wt | wt | wt | wt |  |  |  |  |
| MD-46 | *mucinous* | wt | wt | wt | wt |  |  |  |  |
| MD-47 | *mucinous* | wt | wt | wt | wt |  |  |  |  |
| MD-48 | *mucinous* | wt | ne | ne | ne |  |  |  |  |
| MD-49 | *mucinous* | wt | mutated | wt | wt |  |  |  |  |
| MD-50 | *mucinous* | wt | wt | wt | wt |  |  |  |  |
| MD-51 | *mucinous* | wt | mutated | wt | wt |  |  |  |  |
| MD-52 | *mucinous* | mutated | mutated | wt | ne |  |  |  |  |
| MD-53 | *mucinous* | wt | wt | wt | ne |  |  |  |  |
| MD-54 | *mucinous* | mutated | wt | wt | wt |  |  |  |  |
| MD-55 | *mucinous* | mutated | wt | wt | wt |  |  |  |  |
| LCCH-584 | *mucinous* | mutated | mutated | wt | wt |  |  |  |  |
| LCCH01-351 | *mucinous* | mutated | wt | wt | wt |  |  |  |  |
| LCCH01-556 | *mucinous* | wt | wt | wt | wt |  |  |  |  |
| LCCH01-480 | *mucinous* | wt | wt | wt | wt |  |  |  |  |
| LCCH01-93 | *mucinous* | wt | mutated | wt | wt |  |  |  |  |
| LCCH01-175 | *mucinous* | wt | mutated | wt | wt |  |  |  |  |
| LCCH01-17 | non-mucinous | wt | wt | wt | wt |  |  |  |  |
| LCCH01-18 | non-mucinous | wt | wt | wt | wt |  |  |  |  |
| LCCH01-88 | non-mucinous | wt | wt | wt | wt |  |  |  |  |
| LCCH01-92 | non-mucinous | wt | wt | wt | wt |  |  |  |  |
| LCCH01-97 | non-mucinous | wt | wt | mutated | wt |  |  |  |  |
| LCCH01-106 | non-mucinous | wt | wt | wt | wt |  |  |  |  |
| LCCH01-110 | non-mucinous | wt | wt | wt | wt |  |  |  |  |
| LCCH01-111 | non-mucinous | wt | wt | wt | wt |  |  |  |  |
| LCCH01-116 | non-mucinous | wt | mutated | wt | wt |  |  |  |  |
| LCCH01-121 | non-mucinous | wt | ne | mutated | wt |  |  |  |  |
| LCCH01-139 | non-mucinous | wt | wt | wt | wt |  |  |  |  |
| LCCH01-162 | non-mucinous | wt | wt | wt | wt |  |  |  |  |
| LCCH01-173 | non-mucinous | wt | wt | wt | wt |  |  |  |  |
| LCCH01-179 | non-mucinous | wt | mutated | wt | ne |  |  |  |  |
| LCCH01-191 | non-mucinous | wt | wt | wt | wt |  |  |  |  |
| LCCH01-192 | non-mucinous | wt | wt | wt | wt |  |  |  |  |
| LCCH01-208 | non-mucinous | mutated | wt | ne | wt |  |  |  |  |
| LCCH01-258 | non-mucinous | wt | wt | wt | wt |  |  |  |  |
| LCCH01-266 | non-mucinous | wt | wt | ne | wt |  |  |  |  |
| LCCH01-143 | non-mucinous | wt | mutated | wt | wt |  |  |  |  |
| LCCH01-159 | non-mucinous | wt | wt | wt | wt |  |  |  |  |
| LCCH01-180 | non-mucinous | wt | wt | ne | wt |  |  |  |  |
| LCCH01-516 | non-mucinous | wt | wt | wt | wt |  |  |  |  |
| LCCH01-555 | non-mucinous | wt | wt | mutated | ne |  |  |  |  |
| LCCH01-557 | non-mucinous | wt | wt | wt | wt |  |  |  |  |
| LCCH01-559 | non-mucinous | wt | wt | wt | wt |  |  |  |  |
| LCCH01-562 | non-mucinous | wt | wt | wt | wt |  |  |  |  |
| LCCH01-565 | non-mucinous | wt | wt | wt | wt |  |  |  |  |
| LCCH01-473 | non-mucinous | wt | mutated | wt | wt |  |  |  |  |
| LCCH01-608 | non-mucinous | wt | mutated | wt | wt |  |  |  |  |
| LCCH01-568 | non-mucinous | wt | wt | wt | wt |  |  |  |  |
| LCCH01-524 | non-mucinous | wt | wt | wt | wt |  |  |  |  |
| LCCH01-490  MD-42 | non-mucinous  non-mucinous | wt  wt | Wt  wt | Wt  wt | wt  mutated |  |  |  |  |
|  |  |  |  |  |  |  |  |  |  |
| wt, wild-type; ne, not evaluated | |  |  |  |  |  |  |  |  |
